# Time-Kill Evaluation of Antibiotic Combinations Containing Ceftazidime-Avibactam against Extensively Drug-Resistant Pseudomonas aeruginosa and Their Potential Role against Ceftazidime-Avibactam-Resistant Isolates

**DOI:** 10.1128/spectrum.00585-21

**Published:** 2021-07-28

**Authors:** María M. Montero, Sandra Domene Ochoa, Carla López-Causapé, Sonia Luque, Luisa Sorlí, Núria Campillo, Inmaculada López Montesinos, Eduardo Padilla, Núria Prim, Ariadna Angulo-Brunet, Santiago Grau, Antonio Oliver, Juan P. Horcajada

**Affiliations:** a Infectious Diseases Service, Hospital del Mar, Infectious Pathology and Antimicrobials Research Group (IPAR), Institut Hospital del Mar d’Investigacions Mèdiques (IMIM), Universitat Autònoma de Barcelona (UAB), CEXS-Universitat Pompeu Fabra Barcelona, Barcelona, Spain; b Servicio de Microbiología y Unidad de Investigación, Hospital Son Espases, IdISBa, Palma de Mallorca, Spain; c Pharmacy Service, Hospital del Mar, Barcelona, Spain; d Laboratori de Referència de Catalunya, Barcelona, Spain; e Psychology and Education Science Studies, Universitat Oberta de Catalunya, Barcelona, Spain; University of Arizona/Banner Health

**Keywords:** ceftazidime-avibactam, colistin, aztreonam, amikacin, combination therapy, *Pseudomonas aeruginosa*

## Abstract

Ceftazidime-avibactam (CZA) has emerged as a promising solution to the lack of new antibiotics against Pseudomonas aeruginosa infections. Data from *in vitro* assays of CZA combinations, however, are scarce. The objective of our study was to perform a time-kill analysis of the effectiveness of CZA alone and in combination with other antibiotics against a collection of extensively drug-resistant (XDR) P. aeruginosa isolates. Twenty-one previously characterized representative XDR P. aeruginosa isolates were selected. Antibiotic susceptibility was tested by broth microdilution, and results were interpreted using CLSI criteria. The time-kill experiments were performed in duplicate for each isolate. Antibiotics were tested at clinically achievable free-drug concentrations. Different treatment options, including CZA alone and combined with amikacin, aztreonam, meropenem, and colistin, were evaluated to identify the most effective combinations. Seven isolates were resistant to CZA (MIC ≥ 16/4 mg/liter), including four metallo-β-lactamase (MBL)-carrying isolates and two class A carbapenemases. Five of them were resistant or intermediate to aztreonam (MIC ≥ 16 mg/liter). Three isolates were resistant to amikacin (MIC ≥ 64 mg/liter) and one to colistin (MIC ≥ 4 mg/liter). CZA monotherapy had a bactericidal effect in 100% (14/14) of the CZA-susceptible isolates. Combination therapies achieved a greater overall reduction in bacterial load than monotherapy for the CZA-resistant isolates. CZA plus colistin was additive or synergistic in 100% (7/7) of the CZA-resistant isolates, while CZA plus amikacin and CZA plus aztreonam were additive or synergistic in 85%. CZA combined with colistin, amikacin, or aztreonam was more effective than monotherapy against XDR P. aeruginosa isolates. A CZA combination could be useful for treating XDR P. aeruginosa infections, including those caused by CZA-resistant isolates.

**IMPORTANCE** The emergence of resistance to antibiotics is a serious public health problem worldwide and can be a cause of mortality. For this reason, antibiotic treatment is compromised, and we have few therapeutic options to treat infections. The main goal of our study is to search for new treatment options for infections caused by difficult-to-treat resistant germs. Pseudomonas aeruginosa is a Gram-negative bacterium distributed throughout the world with the ability to become resistant to most available antibiotics. Ceftazidime-avibactam (CZA) emerged as a promising solution to the lack of new antibiotics against infections caused by P. aeruginosa strains. This study intended to analyze the effect of CZA alone or in combination with other available antibiotics against P. aeruginosa strains. The combination of CZA with other antibiotics could be more effective than monotherapy against extensively drug-resistant P. aeruginosa strains.

## INTRODUCTION

New therapeutic options for multidrug-resistant (MDR) and extensively drug-resistant (XDR) Pseudomonas aeruginosa infections are required to overcome the growing problem of antimicrobial resistance. According to the U.S. Centers for Disease Control and Prevention, XDR P. aeruginosa is a “serious threat” to human health, and resistance is on the rise (1). This bacterium has a nonclonal epidemic population structure (2) and can develop antibiotic resistance through several mechanisms. XDR P. aeruginosa high-risk clones are disseminated in hospitals around the world (2) and pose a major public health problem because of limited treatment options and rising costs. Sequence type 111 (ST111) and ST235 are the predominant high-risk clones worldwide, but in Spain, the predominant clone is ST175 (2). High-risk clones are frequently responsible for nosocomial infections and are associated with the acquisition of horizontally transferable beta-lactamases and resistance mechanisms through chromosomal mutations (2, 3).

The problem of increasing antimicrobial resistance is compounded by a dwindling supply of new drugs. Given the few antibiotics in the clinical pipeline before 2010, the treatment options for XDR P. aeruginosa infections were suboptimal and consisted largely of antibiotics with a narrow therapeutic window and high toxicity (aminoglycosides, polymyxins) or unpredictable pharmacokinetics (colistin), yielding poor patient outcomes (4–7).

Ceftazidime-avibactam (CZA) was approved by the U.S. Food and Drug Administration in 2015 and was the first β-lactam combination to provide broad coverage against XDR Gram-negative pathogens, including P. aeruginosa (8). Few studies, however, have examined the effectiveness of CZA against infections caused by XDR P. aeruginosa high-risk clones. An *in vitro* study of a large collection of P. aeruginosa strains reported a CZA resistance rate of 2.9% (9). Most studies, however, have reported higher rates, up to 18% in some cases (10) and over 50% when XDR strains are involved (11, 12). Strains carrying metallo-β-lactamases (MBLs) have the highest resistance rates (>95%) as they are resistant to CZA, and CZA is not expected to be efficacious against these strains (13).

The use of CZA to treat P. aeruginosa infections caused by XDR high-risk clones may be clinically more effective and less toxic than colistin, which is often the only option available (14). However, given the high risk for the emergence of CZA-resistant mutants, it is paramount to monitor their selection during treatment and to evaluate associated risk factors. Combination therapy is a useful strategy for achieving maximum antimicrobial activity against various resistant organisms and for preventing antibiotic resistance (15). *In vitro* experiments have shown synergy for certain antipseudomonal antibiotics against MDR P. aeruginosa (5, 15–20). *In vitro* studies evaluating the activity of CZA combined with other antibiotics against P. aeruginosa, however, are lacking, and only few reports covering a small number of isolates have been published (21).

The aim of this study was to perform a comprehensive time-kill analysis of CZA alone or in combination with standard antipseudomonal antibiotics against a representative collection of the most common resistance mechanisms and XDR P. aeruginosa clones, including high-risk clones.

## RESULTS

### Antimicrobial susceptibility testing.

The antibiotic susceptibility profiles and previously characterized antibiotic resistance mechanisms of the 21 XDR P. aeruginosa isolates are shown in Table 1. Seven isolates were resistant to both CZA (MIC ≥ 16/4 mg/liter) and meropenem (MIC ≥ 8 mg/liter), and of these, four were resistant and one was intermediate to aztreonam (MIC ≥ 16 mg/liter), three were resistant to amikacin (MIC ≥ 64 mg/liter), and one was resistant to colistin (MIC ≥ 4 mg/liter). Six of the seven CZA-resistant isolates harbored carbapenemases belonging to Ambler class A or B and had OprD deficiency, except for one, and two of them showed AmpC hyperproduction.

**TABLE 1:** Antibiotic susceptibility profile and resistance mechanisms of the 21 XDR P. aeruginosa isolates^*a*^

Isolate	ST	Acquired β- lactamase(s)	AmpC hyperproduction	OprD deficiency	MIC (mg/liter)				
					AMK	ATM	MEM	CST	CZA
04-017	111	OXA-46	Yes	No	4	64	32	2	8
04-025	175		Yes	Yes	4	16	16	1	4
10-023	175		Yes	Yes	4	16	16	2	4
06-014	179	OXA-10	Yes	Yes	8	16	32	2	4
12-003	244		Yes	Yes	8	32	32	2	4
09-011	274		Yes	Yes	128	64	32	1	4
09-007	313		Yes	Yes	8	32	16	2	4
10-017	395		Yes	No	4	32	8	2	4
06-035	455		Yes	No	<2	64	>32	0.5	8
10-019	2221		Yes	Yes	<2	64	32	2	8
06-025	2534		Yes	Yes	<2	64	8	2	8
06-027	2535		Yes	No	8	32	8	2	4
06-001	2536		Yes	Yes	8	64	32	2	8
09-012	175		Yes	Yes	8	64	16	2	8
10-009	111	VIM-2	Yes	Yes	32	>128	>32	4	>32
07-016	175	GES-5	No	Yes	16	16	>32	2	32
12-012	175	VIM-20, OXA-2	No	Yes	16	8	>32	2	32
07-004	235	GES-19, OXA-2	No	Yes	128	128	>32	2	>32
06-042	235	VIM-47	No	No	64	32	>32	2	32
01-008	253	VIM-1	No	Yes	8	4	>32	2	>32
10-021	2533		Yes	Yes	<2	64	32	1	16

a MICs (mg/liter) of the following antibiotics tested in this study are shown: amikacin (AMK), aztreonam (ATM), meropenem (MEM),colistin (CST), and ceftazidime-avibactam (CZA). CZA-resistant isolates are highlighted in gray.

### Time-kill studies.

**FIG 1:**
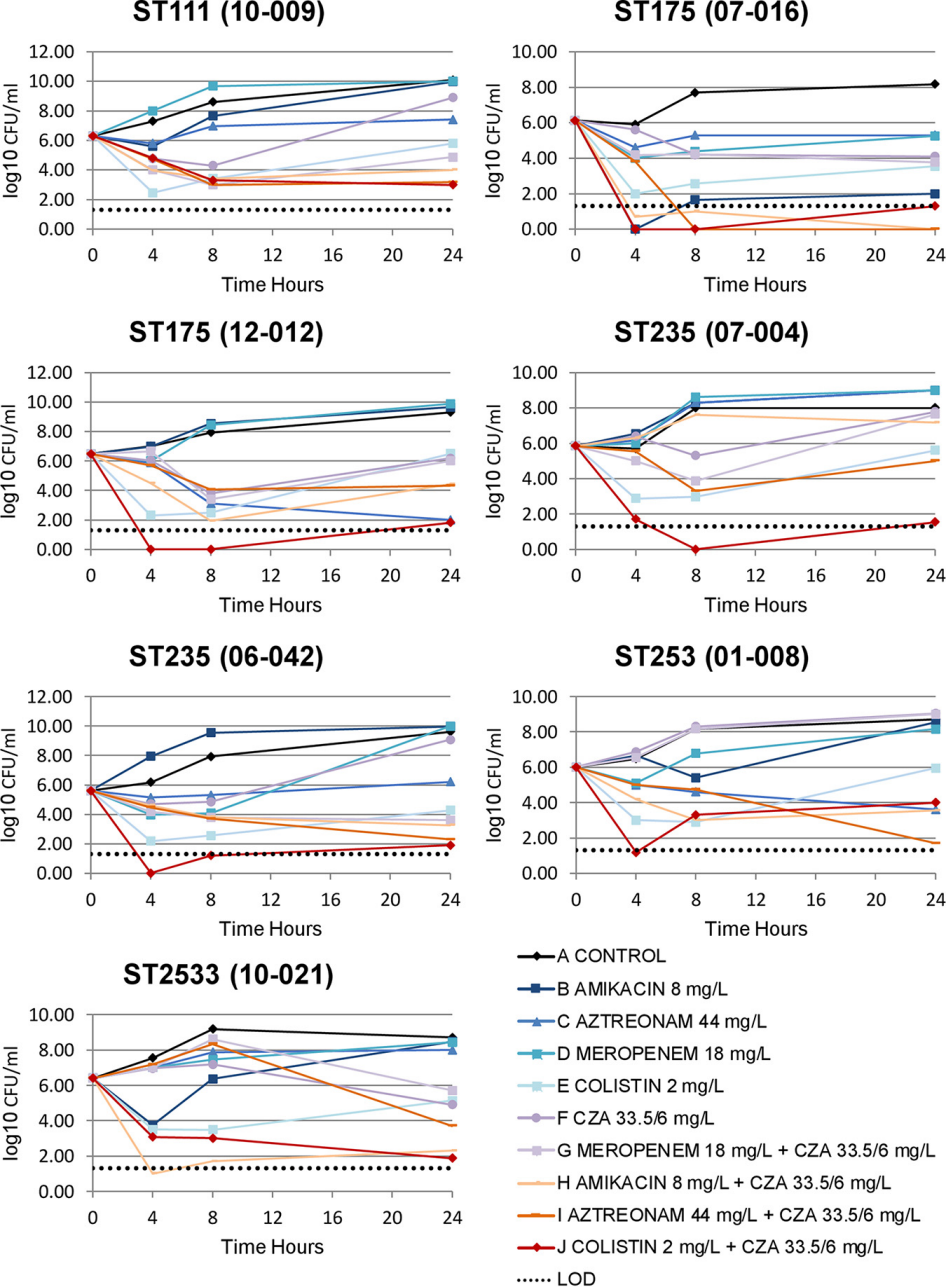
Bacterial load (log_10_ CFU/ml) over 24 h in the seven CZA-resistant XDR P. aeruginosa isolates for each antibiotic regimen. LOD, lower limit of detection.

Bacterial growth without antibiotic reached 8 to 9 log_10_ CFU/ml at 24 h for all isolates. The results of the time-kill experiments for the 21 XDR P. aeruginosa isolates are shown in Table S1 in the supplemental material. The mean bacterial loads (log_10_ CFU/ml) over 24 h for the seven CZA-resistant XDR P. aeruginosa isolates treated with each antibiotic regimen are shown in Fig. 1. Table 2 shows the synergistic and additive effects of each combination against CZA-susceptible and CZA-resistant isolates. Table S2 shows the time-kill results (log difference at 24 h) for each antibiotic compared with the control and for each antibiotic combination compared with each antibiotic.

**TABLE 2:** Synergistic and additive effects of each antibiotic combination against CZA-susceptible and CZA-resistant P. aeruginosa isolates^*a*^

Antibiotic combination	% of isolates					
	CZA susceptible			CZA resistant		
	Synergy	Additivity	Total	Synergy	Additivity	Total
AMK+CZA	8	3	78.6	5	1	85.7
ATM+CZA	2	4	42.9	4	2	85.7
MEM+CZA	1	1	14.3	2	0	28.6
CST+CZA	6	2	57.1	6	1	100.0

a AMK, amikacin; ATM, aztreonam; MEM, meropenem; CST, colistin; CZA, ceftazidime-avibactam.

Single antibiotics (aztreonam, meropenem, colistin, amikacin) were not bactericidal against any of the isolates at 24 h. Despite this, when compared with the control, all single antibiotics resulted in fewer bacteria than the control (*F*_4, 64_ = 8.7, *P* < 0.001; amikacin dif = −1.34, *t* = −2.5, *P* = 0.02; aztreonam dif = −1.43, *t* = −2.63, *P* = 0.01; meropenem dif = −1.42, *t* = −2.62, *P* = 0.01; colistin dif = −3.18, *t* = −5.87, *P* < 0.001).

CZA monotherapy was bactericidal against all the CZA-susceptible isolates, with a mean reduction of 3.19 log_10_ CFU/ml. In a comparison of the effects of the combination of CZA with other antibiotics, we found differences (*F*_4, 65_ = 11.08, *P* < 0.001). CZA plus amikacin (dif = −1.74, *t* = −3.58, *P* < 0.001) and CZA plus colistin (dif = −1.59, *t* = −3.25, *P* = 0.001) achieved a mean reduction of >4 log_10_ CFU/ml in the same isolates. The best combination against the CZA-susceptible isolates was CZA plus amikacin, which was synergistic or additive in approximately 80% of cases. On the other hand, no differences between CZA alone and CZA with aztreonam were found (dif = −0.48, *t* = −0.99, *P* = 0.33). Furthermore, combining CZA with meropenem increased the number of bacteria in comparison with CZA alone (dif = 1.02, *t* = 1.09, *P* = 0.04).

CZA combination therapies achieved a higher overall reduction in bacterial load than any of the treatments in isolation for the seven CZA-resistant isolates (*F*_1, 61_ = 33.92, *P* < 0.001). The log_10_ CFU/ml mean for the treatments in isolation was 0.94, and combining treatments reduced that mean to 3.44 (*t* = −5.82, *P* < 0.001). Hence, the mean reduction was 4.4 log_10_ CFU/ml for CZA plus colistin, amikacin, or aztreonam. As can be seen in Table 2, CZA plus colistin was either additive or synergistic in 100% of cases, while CZA plus amikacin or aztreonam was additive or synergistic in 85% of cases. The combination of CZA with aztreonam was effective against three of the four MBL-carrying isolates and against the two isolates that harbored class A carbapenemases.

## DISCUSSION

We investigated the use of CZA alone or in combination with four antibiotics to assess the potential synergistic effects against XDR P. aeruginosa. As expected, a bactericidal effect was observed for CZA monotherapy in all the CZA-susceptible P. aeruginosa isolates, which had AmpC hyperproduction and/or OprD deficiency. To preserve the effectiveness of CZA, its clinical use should be avoided in naturally resistant strains and in those carrying MBLs and certain class D β-carbapenemases (22). Combination therapy has an important role in these clinical scenarios, and CZA combined with other antibacterial agents should be considered.

CZA resistance has already been described in Gram-negative bacilli. β-Lactamase-related mutations are the main mechanism behind CZA resistance in *Enterobacterales*. Recent reports suggest that the development of different resistance mechanisms within the course of treatment (e.g., mutations in KPC-encoding genes) might threaten the effectiveness of CZA (23, 24), a phenomenon that could be further complicated by horizontal spread (25). The development of CZA resistance during treatment of P. aeruginosa infections is frequently due to the selection of mutations in the AmpC β-lactamase structure, which are associated with coresistance with ceftolozane-tazobactam (16). Other contributory factors might be diminished outer membrane permeability and/or overexpression of efflux pumps (26). High-level resistance to CZA might also be due to MBL acquisition (27). Overall, six of the seven CZA-resistant isolates in our study harbored acquired β-lactamases, including several MBLs (VIM type) and a serine carbapenemase.

Little has been published on antibiotic combinations containing CZA, especially in the context of XDR P. aeruginosa isolates. Combination therapy with CZA plus aztreonam, amikacin, colistin, fosfomycin, and meropenem was recently evaluated in MDR Klebsiella pneumoniae *and* P. aeruginosa strains, but none of the isolates carried MBLs and few time-kill curves were analyzed (28). A synergistic effect was also reported for the combined use of CZA and colistin against MDR P. aeruginosa strains, including those resistant to colistin (29). In the present study, the combination of CZA with colistin showed a synergistic or additive effect against all the CZA-resistant P. aeruginosa isolates, including a colistin-resistant strain. Synergy was also observed against 85% of these isolates when CZA was combined with amikacin or aztreonam. In the combination of CZA with colistin, several bacterial isolates reached bacterial eradication at 4 and 8 h but then showed a little regrowth at 24 h. The phenomenon of bacterial regrowth could be due to either a loss of functionality of these antibiotics or selection of resistant isolates. Presumably, the latter could include selection of preexisting resistant subpopulations, *de novo* mutations, adaptive resistance, or formation of persistent cells (30). Further studies are required in order to evaluate these possibilities.

A double β-lactam strategy has been tested against carbapenemase-producing enterobacterial isolates in which CZA combined with meropenem or imipenem showed synergy against certain KPC-producing K. pneumoniae strains (31). In our study, however, CZA plus meropenem was the only combination to show no synergistic or additive activity against most XDR P. aeruginosa isolates. This could be because nonenzymatic mechanisms, alongside acquired β-lactamases, may have contributed to high meropenem MICs in the CZA-resistant isolates.

As mentioned, CZA is not active against MBL-bearing strains (22). The addition of aztreonam might overcome this resistance, as MBLs are known to have a weak hydrolysis capacity against aztreonam (32, 33). Combination therapy with ceftazidime and aztreonam may also be beneficial due to the simultaneous inhibition of multiple penicillin-binding proteins (34). Additionally, CZA plus aztreonam could exert an independent effect by acting on the “divisome” of Gram-negative bacteria (27). A recent report based on time-kill experiments with five P. aeruginosa isolates resistant to both CZA and aztreonam found that the combined use of the antibiotics had a synergistic effect and restored bactericidal activity in four of the isolates (21). In our study, this combination was effective against three of the four MBL-carrying isolates.

This study had some limitations. Our results are based on short *in vitro* assays with minimal antibiotic exposure compared with other pharmacokinetic/pharmacodynamic studies. Since these results are not representative of clinical guidelines for the administration of most antibiotics, they must be validated in *in vivo* experiments (35). The experimental design of this type of study does not allow identification of mechanisms of interactions or taking the emergence of resistance into consideration. A strength of our study is that our results are based on a large number of time-kill assays and show evidence of synergistic or additive effects in a considerable proportion of cases.

In conclusion, CZA is effective against XDR P. aeruginosa isolates both alone and in combination with other antibiotics. Combination regimens featuring CZA may be a good option against infections caused by these difficult-to-treat bacteria. Our data support the potential use of CZA in combination with amikacin, aztreonam, and colistin against XDR P. aeruginosa isolates, including CZA-resistant isolates and prevalent high-risk clones. These findings may help identify strategies to improve the clinical management of XDR P. aeruginosa infections using currently available drugs.

## MATERIALS AND METHODS

### Bacterial isolates and resistance mechanisms.

We studied 21 XDR P. aeruginosa clinical isolates which had been previously collected by our group as a part of the COLIMERO trial, a multicenter Spanish trial involving the molecular characterization of 150 XDR P. aeruginosa isolates from nine Spanish hospitals using pulsed-field gel electrophoresis, multilocus sequence typing, and whole-genome sequencing (3). The 21 isolates were representative of the clones and the most prevalent and relevant resistance mechanisms detected in the trial, namely, chromosomal mutations (AmpC hyperproduction and OprD inactivation) and horizontally acquired enzymes, including several MBLs and class A carbapenemases.

### Antibiotics.

The antipseudomonal antibiotics used in the experiments were amikacin, aztreonam, colistin, meropenem (Sigma-Aldrich), and CZA (Pfizer). The antibiotics were chosen based on the mechanism of action and availability in the hospital’s pharmacy. Antibiotic solutions were prepared according to CLSI guidelines (36). Antibiotic concentrations for time-kill experiments were based on area-under-the-curve (AUC) serum levels: for amikacin, 1 g every 24 h (q24h), with an area under the concentration-time curve for 24 h (AUC_24_) of 196 μg · h/ml (37, 38); for aztreonam, 2 g q8h, with an AUC_24_ of 1,050 μg · h/ml (39); for meropenem, 2 g q8h, with an AUC_24_ of 425 μg · h/ml (40); for colistin, 4.5 MIU (million International units) q12h, with an AUC_24_ of 50 μg · h/ml (41, 42); for CZA, 2 g q8h, with an AUC_24_ of 800 μg · h/ml (43); and for avibactam, 2 g q8h, with an AUC_24_ of 147 μg · h/ml (43).

### Antibiotic susceptibility testing.

The susceptibility profiles of the XDR isolates were obtained from the COLIMERO trial (3). Antimicrobial susceptibility was tested using broth microdilution and agar dilution methods with cation-adjusted Mueller-Hinton II broth (CAMHB) and Mueller-Hinton (MH) agar media, according to the CLSI guidelines (36). Ceftazidime susceptibility testing was conducted alone and in combination with a fixed avibactam concentration (4 mg/liter).

### Time-kill experiments.

Time-kill studies were performed to analyze the activity of the selected antibiotics alone and in combination with CZA at clinically achievable free-drug concentrations. All experiments were performed in duplicate. An overnight culture of isolate was diluted with CAMHB and further incubated at 37°C for an hour to reach early log-phase growth. The bacterial suspension was diluted with CAMHB according to the absorbance at 630 nm. The magnitudes of absorbance ranged from 0.2 to 0.4. Sterile 50-ml conical flasks were used with 30 ml of CAMHB supplemented with the corresponding antibiotics. The final bacterial inoculum was approximately 6 to 7 log_10_ CFU/ml per flask. Flasks were incubated at 37°C in a shaker water bath for 24 h. Samples were collected at 0, 4, 8, and 24 h to measure bacterial growth. A 1-ml aliquot was obtained from each flask at each time point, centrifuged at 13,000 rpm for 3 min, and reconstituted with sterile saline solution to its original volume to minimize drug carryover. Serial decimal dilutions in CAMHB were performed; MH agar plates were inoculated (200 μl per plate) and incubated in a humidified incubator (37°C) for 18 to 24 h. Bacterial colonies for each sample were counted after overnight incubation. The bacterial density from the original sample was calculated based on the dilution factor. The limit of detection (LOD) was 1.3 log_10_ CFU/ml.

Apart from describing the results, in order to assess the effect of monotherapy and of the antibiotic combinations, we performed a series of regression analyses in which we entered the log difference in 24 h as dependent variable and each antibiotic regimen as independent variable. We checked for the application conditions of the regression, and all the conditions were met (normality of the residuals [assessed with Shapiro-Wilk’s test] and homoscedasticity [assessed with the Breusch-Pagan test]).

### Pharmacodynamic time-kill parameters.

The results of the time-kill experiments were read at the different time points (0, 4, 8, and 24 h). Bactericidal activity was defined as a ≥3-log_10_ CFU/ml reduction, synergy as a ≥2-log_10_ CFU/ml reduction for a given combination compared with the most active single agent, additivity as a 1- to 2-log_10_ CFU/ml reduction in the final colony count for the combination compared with the most active single agent, and antagonism as a regrowth to ≥1-log_10_ CFU/ml for the combination compared with the least active single agent (44, 45). In addition to the aforementioned relevance criteria, we applied regression analysis to determine if the difference in log_10_ was statistically significant.
