# Supplementary material for: Time-Kill Evaluation of Antibiotic Combinations Containing Ceftazidime-Avibactam against Extensively Drug-Resistant Pseudomonas aeruginosa and Their Potential Role against Ceftazidime-Avibactam-Resistant Isolates
Source: Microbiol Spectr. 2021 Jul 28;9(1):10.1128/spectrum.00585-21. doi: 10.1128/spectrum.00585-21 (PMC8552783; doi:10.1128/spectrum.00585-21)
Supplement: SUPPLEMENTAL FILE 1 — Supplemental material. Download SPECTRUM00585-21_Supp_1_seq4.pdf, PDF file, 0.3 MB. [file spectrum00585-21_supp_1_seq4.pdf]

Table S1. Time-kill experiments with 21 XDR *P. aeruginosa* isolates.

Isolate	ST	0h	Atb	8h	24h	Δ24h	Synergy
04-017	**111**	6,08	Control	7,70	9,30	3,2	
			AMK	3,19	7,65	1,6	
			ATM	5,31	7,88	1,8	
			MEM	5,76	9,00	2,9	
			CST	3,55	5,51	-0,6	
			CZA	3,54	2,93	-3,2	
			AMK+CZA	0,00	0,00	-6,1	-2,9
			ATM+CZA	3,74	3,30	-2,8	0,4
			MEM+CZA	3,30	5,00	-1,1	2,1
			CST+CZA	1,98	2,39	-3,7	-0,5
04-025	**175**	6,85	Control	7,18	8,90	2,1	
			AMK	3,50	6,42	-0,4	
			ATM	4,33	5,60	-1,3	
			MEM	3,21	5,18	-1,7	
			CST	2,63	6,32	-0,5	
			CZA	3,93	3,90	-3,0	
			AMK+CZA	1,40	4,88	-2,0	1,0
			ATM+CZA	3,93	1,90	-5,0	-2,0
			MEM+CZA	3,48	3,98	-2,9	-1,2
			CST+CZA	1,81	1,88	-5,0	-2,0
10-023	**175**	6,7	Control	9,18	9,60	2,9	
			AMK	3,70	6,30	-0,4	
			ATM	6,18	4,00	-2,7	
			MEM	4,5	7,93	1,2	
			CST	3,69	8,09	1,4	
			CZA	5,04	2,74	-4,0	
			AMK+CZA	2,13	1,54	-5,2	-1,2
			ATM+CZA	3,98	2,00	-4,7	-0,7
			MEM+CZA	3,06	2,00	-4,7	-0,7
			CST+CZA	1,30	2,04	-4,7	-0,7
06-014	**179**	6,4	Control	8,00	9,10	2,7	
			AMK	4,54	5,90	-0,5	
			ATM	3,00	6,74	0,3	
			MEM	5,86	8,27	1,9	
			CST	1,85	4,82	-1,6	
			CZA	2,60	3,41	-3,0	
			AMK+CZA	1,00	1,00	-5,4	-2,4
			ATM+CZA	2,18	1,88	-4,5	-1,5
			MEM+CZA	3,00	3,60	-2,8	0,2
			CST+CZA	0,00	0,00	-6,4	-3,4
12-003	**244**	6,86	Control	8,48	9,30	2,4	
			AMK	7,70	9,18	2,3	
			ATM	7,93	9,18	2,3	
			MEM	9,42	9,66	2,8	
			CST	5,28	6,9	0,0	
			CZA	4,74	3,81	-3,1	
			AMK+CZA	2,18	1,81	-5,0	-2,0
			ATM+CZA	3,60	2,30	-4,6	-1,5
			MEM+CZA	6,00	7,40	0,5	3,6
			CST+CZA	0,00	2,80	-4,1	-1,0
09-011	**274**	6,4	Control	8,18	8,70	2,3	
			AMK	8,10	9,35	3,0	
			ATM	7,60	9,16	2,8	
			MEM	5,57	9,8	3,4	
			CST	1,71	3,8	-2,6	
			CZA	3,60	2,18	-4,2	
			AMK+CZA	2,00	0,00	-6,4	-2,2
			ATM+CZA	4,04	3,43	-3,0	1,3
			MEM+CZA	3,30	3,18	-3,2	1,0
			CST+CZA	0,00	0,00	-6,4	-2,2
09-007	**313**	6,9	Control	7,74	7,93	1,0	
			AMK	7,35	8,04	1,1	
			ATM	7,18	7,81	0,9	
			MEM	3,85	5,39	-1,5	
			CST	3,56	7,68	0,8	
			CZA	4,00	3,90	-3,0	
			AMK+CZA	3,85	3,88	-3,0	0,0
			ATM+CZA	6,06	6,48	-0,4	2,6
			MEM+CZA	4,32	4,30	-2,6	0,4
			CST+CZA	3,00	3,78	-3,1	-0,1

Isolate	ST	0h	Atb	8h	24h	Δ24h	Synergy
10-017	**395**	6,74	Control	8,74	9,30	2,6	
			AMK	5,00	8,48	1,7	
			ATM	7,19	9,00	2,3	
			MEM	3,95	9,10	2,4	
			CST	3,15	4,50	-2,2	
			CZA	4,88	3,70	-3,0	
			AMK+CZA	2,60	1,65	-5,1	-2,0
			ATM+CZA	4,33	2,60	-4,1	-1,1
			MEM+CZA	5,00	6,48	-0,3	2,8
			CST+CZA	2,11	2,70	-4,0	-1,0
06-035	**455**	6,96	Control	8,60	9,18	2,2	
			AMK	1,74	5,60	-1,4	
			ATM	5,06	8,48	1,5	
			MEM	3,8	8,64	1,7	
			CST	3,45	6	-1,0	
			CZA	3,70	4,00	-3,0	
			AMK+CZA	1,74	1,60	-5,4	-4,0
			ATM+CZA	3,74	2,00	-5,0	-2,0
			MEM+CZA	3,48	3,65	-3,3	-0,3
			CST+CZA	0,00	2,00	-5,0	-4,0
10-019	**2221**	6,99	Control	8,00	8,65	1,7	
			AMK	3,88	7,18	0,2	
			ATM	8,59	9,65	2,7	
			MEM	5,51	8,9	1,9	
			CST	2,44	6,35	-0,6	
			CZA	3,30	4,00	-3,0	
			AMK+CZA	2,40	2,60	-4,4	-1,4
			ATM+CZA	2,65	3,00	-4,0	-1,0
			MEM+CZA	2,30	7,15	0,2	3,1
			CST+CZA	2,54	1,65	-5,3	-2,4
06-025	**2534**	6,98	Control	8,18	9,70	2,7	
			AMK	3,18	8,18	1,2	
			ATM	7,70	7,74	0,8	
			MEM	4,15	5,05	-1,9	
			CST	3,66	5,54	-1,4	
			CZA	4,48	4,00	-3,0	
			AMK+CZA	0,00	1,00	-6,0	-3,0
			ATM+CZA	3,65	2,00	-5,0	-2,0
			MEM+CZA	2,93	1,90	-5,1	-3,2
			CST+CZA	0,00	0,00	-7,0	-5,5
06-027	**2535**	6,48	Control	9,06	9,65	3,2	
			AMK	7,74	9,00	2,5	
			ATM	7,33	6,74	0,3	
			MEM	3,94	3,80	-2,7	
			CST	3,92	4,17	-2,3	
			CZA	3,78	2,48	-4,0	
			AMK+CZA	2,78	0,70	-5,8	-1,8
			ATM+CZA	5,04	2,70	-3,8	0,2
			MEM+CZA	5,19	3,70	-2,8	1,2
			CST+CZA	3,00	1,70	-4,8	-0,8
06-001	**2536**	6,95	Control	8,88	9,54	2,6	
			AMK	5,81	7,48	0,5	
			ATM	7,00	8,02	1,1	
			MEM	5,77	8,56	1,6	
			CST	4,34	6,89	-0,1	
			CZA	8,04	4,00	-3,0	
			AMK+CZA	2,18	1,90	-5,1	-2,1
			ATM+CZA	5,70	5,10	-1,9	1,1
			MEM+CZA	6,20	6,00	-1,0	2,0
			CST+CZA	3,35	2,00	-5,0	-2,0
09-012	**175**	6,84	Control	8,00	9,18	2,3	
			AMK	8,18	9,60	2,8	
			ATM	7,40	7,18	0,3	
			MEM	7,30	8	1,2	
			CST	5,32	6	-0,8	
			CZA	6,08	3,89	-3,0	
			AMK+CZA	2,70	1,90	-4,9	-2,0
			ATM+CZA	3,20	3,40	-3,4	-0,5
			MEM+CZA	3,74	4,90	-1,9	1,0
			CST+CZA	2,81	3,78	-3,1	-0,1

Isolate	ST	0h	Atb	8h	24h	Δ24h	Synergy
10-009	**111**	6,3	Control	8,60	10,10	3,8	
			AMK	7,65	9,98	3,7	
			ATM	6,97	7,41	1,1	
			MEM	9,69	10,01	3,7	
			CST	3,42	5,81	-0,5	
			CZA	4,30	8,90	2,6	
			AMK+CZA	3,40	4,00	-2,3	-4,9
			ATM+CZA	3,00	3,18	-3,1	-4,2
			MEM+CZA	3,00	4,88	-1,4	-4,0
			CST+CZA	3,30	3,00	-3,3	-2,8
07-016	**175**	6,13	Control	7,70	8,18	2,0	
			AMK	1,65	2,00	-4,1	
			ATM	5,30	5,30	-0,8	
			MEM	4,39	5,26	-0,9	
			CST	2,56	3,55	-2,6	
			CZA	4,19	4,10	-2,0	
			AMK+CZA	1,00	0,00	-6,1	-2,0
			ATM+CZA	0,00	0,00	-6,1	-2,0
			MEM+CZA	4,24	3,78	-2,4	-0,3
			CST+CZA	0,00	1,30	-4,8	-2,2
12-012	**175**	6,48	Control	7,93	9,30	2,8	
			AMK	8,54	9,65	3,2	
			ATM	3,11	2,00	-4,5	
			MEM	8,44	9,88	3,4	
			CST	2,48	6,5	0,0	
			CZA	3,81	6,18	-0,3	
			AMK+CZA	1,93	4,42	-2,1	-1,8
			ATM+CZA	4,06	4,32	-2,2	2,3
			MEM+CZA	3,40	6,00	-0,5	-0,2
			CST+CZA	0,00	1,81	-4,7	-4,4
07-004	**235**	6,85	Control	8,00	8,00	1,2	
			AMK	8,30	9,00	2,2	
			ATM	8,30	9,00	2,2	
			MEM	8,61	9	2,2	
			CST	2,97	5,6	-1,3	
			CZA	5,3	7,8	0,9	
			AMK+CZA	7,6	7,2	0,3	-0,6
			ATM+CZA	3,30	5,00	-1,9	-2,8
			MEM+CZA	3,88	7,65	0,8	-0,1
			CST+CZA	0,00	1,54	-5,3	-4,1
06-042	**235**	5,6	Control	7,93	9,60	4,0	
			AMK	9,54	9,97	4,4	
			ATM	5,31	6,21	0,6	
			MEM	4,07	9,98	4,4	
			CST	2,56	4,27	-1,3	
			CZA	4,85	9,06	3,5	
			AMK+CZA	3,81	3,27	-2,3	-5,8
			ATM+CZA	3,70	2,30	-3,3	-3,9
			MEM+CZA	3,78	3,60	-2,0	-5,5
			CST+CZA	1,20	1,90	-3,7	-2,4
01-008	**253**	6	Control	8,18	8,70	2,7	
			AMK	5,40	8,54	2,5	
			ATM	4,60	3,60	-2,4	
			MEM	6,78	8,15	2,2	
			CST	2,9	5,95	0,0	
			CZA	8,30	9,04	3,0	
			AMK+CZA	3,00	3,54	-2,5	-5,0
			ATM+CZA	4,74	1,70	-4,3	-1,9
			MEM+CZA	8,18	9,00	3,0	0,9
			CST+CZA	3,30	4,00	-2,0	-2,0
10-021	**2533**	6,4	Control	9,18	8,70	2,3	
			AMK	6,35	8,48	2,1	
			ATM	7,88	8,00	1,6	
			MEM	7,45	8,43	2,0	
			CST	3,48	5,13	-1,3	
			CZA	7,18	4,90	-1,5	
			AMK+CZA	1,70	2,30	-4,1	-2,6
			ATM+CZA	8,33	3,70	-2,7	-1,2
			MEM+CZA	8,60	5,70	-0,7	0,8
			CST+CZA	3,00	1,88	-4,5	-3,0

Atb, antibiotic; AMK, amikacin; ATM, aztreonam; MEM, meropenem; CST, colistin; CZA, ceftazidime-avibactam.

Bactericidal effects (≥3-log_{10} reduction in CFU/ml after 24 h) are highlighted in green. Synergistic and additive effects (≥2-log_{10} or ≥1-log_{10} reduction in CFU/ml at 24 h with the combination compared with the most active single drug) are highlighted in yellow and orange, respectively. Bacterial loads (log_{10} CFU/ml) are shown for each isolate and antibiotic treatment.

Table S2. Log difference (diff) at 24 h for each antibiotic alone compared with the control, and for each antibiotic combination compared with each antibiotic.

	**CZA-resistant**	**CZA-susceptible**
	**Log diff 24 h**	**Log diff 24 h**
AMK vs control	-0.71	-1.41**
ATM vs control	-3.01*	-1.49**
MEM vs control	-0.27	-1.48*
CST vs control	-3.68**	-3.25***
CZA vs control	-1.80	-5.65***
MEM+CZA vs MEM	-2.87*	-3.15***
MEM+CZA vs CZA	-1.34	1.02*
AMK+CZA vs AMK	-4.70**	-5.99***
AMK+CZA vs CZA	-3.61*	-1.75***
ATM+CZA vs ATM	-3.05*	-4.64***
ATM+CZA vs CZA	-4.25*	-0.48
CST+CZA vs CST	-3.05**	-3.99***
CST+CZA vs CZA	-4.93***	-1.59***

AMK, amikacin; ATM, aztreonam; MEM, meropenem; CST, colistin; CZA, ceftazidime-avibactam.

Note. **p<.001, ‘*’p<.05
